# Supplementary material for: Content-rich biological network constructed by mining PubMed abstracts
Source: BMC Bioinformatics. 2004 Oct 8;5:147. doi: 10.1186/1471-2105-5-147 (PMC528731; doi:10.1186/1471-2105-5-147)
Supplement: Additional File 2 — The original results of the above study (non-essential files are deleted to keep the file size under the limit set by BMC bioinformatics). [file 1471-2105-5-147-S2.bz2 › chilibotAdditionalFile2/dip40/616ID10360578E11000/html/CDC42_WAS.html]

 


 **CDC42** and **WAS** 
  
Found 126 abstracts in PubMed, retrieved 40.  
 

 What does Google say? 
 PDF only 
| .edu only 

---

**Interactive relationship** (e.g. stimulation, inhibition, etc)

**Inhibitory relationship**- We also searched for the  **Cdc42**  V12 target but overexpression of the  **Cdc42**  effector WASP  [ **WAS** ]  did not mimic the inhibition of exocytosis observed in cells transfected with the activated GTPase.  Ref: 11822867 Exp Cell Res, 2002
- We show that Map induced filopodia formation is independent of mitochondrial targeting and is abolished by cellular expression of the  **Cdc42**  inhibitory WASP  [ **WAS** ]  CRIB domain, demonstrating that Map has at least two distinct functions in host cells.  Ref: 12046591 Mol Microbiol, 2002
- Furthermore, overexpression of WASp  [ **WAS** ]  inhibits anterograde transport of MNK, further supporting regulation by the  **Cdc42**  GTPase.  Ref: 12393797 Hum Mol Genet, 2002
- In resting cells, WASP  [ **WAS** ]  exists in a complex with WIP, which inhibits its activation by  **Cdc42** .  Ref: 12504004 Mol Cell, 2002
- WASp  [ **WAS** ]  is capable of forming an auto inhibited conformation, which can be disrupted by binding of  **Cdc42**  and phosphatidylinositol bisphosphate, leading to its activation.  Ref: 12235133 J Biol Chem, 2002
**Stimulatory relationship**- In contrast, secretion elicited by depolarizing K concentrations  **was**  enhanced by the dominant negative mutants RhoA N19, Rac1 N17, and  **Cdc42**  N17 and  **was**  diminished by the constitutively active mutants RhoA V14, Rac1 V12, and  **Cdc42**  V12.  Ref: 11822867 Exp Cell Res, 2002
- In an analogous pathway, direct interaction of  **Cdc42**  with the related protein N WASP  [ **WAS** ]  stimulates actin polymerization.  Ref: 12181570 Nature, 2002
- However, in the presence of the active form of the GTPase  **Cdc42**  Cdc42V12, which is thought to switch WASP  [ **WAS** ]  to an active open conformation, the amount of WASP  [ **WAS** ]  associated with the receptor  **was**  markedly increased.  Ref: 12600272 Biochem J< MedlineTA>Biochem J, 2002
- The Wiskott Aldrich syndrome protein  [ **WAS** ]  WASP  [ **WAS** ]  is an effector of the Rho family GTPase  **Cdc42** , whose activation leads to stimulation of the actin nucleating assembly, Arp2 3 complex.  Ref: 12769846 Mol Cell, 2003
**Neutral relationship**- In contrast, the direct interaction of N WASP  [ **WAS** ]  with the Rho GTPase  **Cdc42**   **was**  not required for reconstitution of vesicle motility.  Ref: 12147689 J Biol Chem, 2002
- In contrast, expression of TC10 Q75L completely disrupted adipocyte cortical actin, which  **was**  specific for TC10, because expression of constitutively active  **Cdc42**   **was**  without effect.  Ref: 12134073 Mol Biol Cell, 2002
- Using bacterial glutathione S transferase  **Cdc42**  fusion proteins and co immunoprecipitation and transient transfection studies,  **Cdc42**   **was**  shown to be an upstream regulator of the exocytotic protein, syntaxin.  Ref: 12135388 Biochemistry, 2002
- Regulation of protein transport from the Golgi complex to the endoplasmic reticulum by  **CDC42**  and N WASP  [ **WAS** ] .  Ref: 11907268 Mol Biol Cell, 2002
- In Vav 1 deficient T cells, WASP  [ **WAS** ]  recruitment proceeds normally, but localized activation of  **Cdc42**  and WASP  [ **WAS** ]  is disrupted.  Ref: 12874226 J ImmunolJ Immunol, 2002
- Melanosome enriched fractions isolated from human melanocytes expressed the  **Cdc42**  effector proteins PAK1 and N WASP  [ **WAS** ]  by western blotting.  Ref: 11896192 J Cell Sci, 2002
- SLP 76 Coordinates Nck Dependent Wiskott Aldrich Syndrome Protein  [ **WAS** ]  Recruitment with Vav 1  **Cdc42**  Dependent Wiskott Aldrich Syndrome Protein  [ **WAS** ]  Activation at the T Cell APC Contact Site.  Ref: 12874226 J ImmunolJ Immunol, 2002
- A human homolog of K08E3.3b is the  **Cdc42**  interacting protein 4 CIP4, a protein involved in  **Cdc42**  and Wiskott Aldrich syndrome protein  [ **WAS** ]  dependent signal transduction.  Ref: 12604778 Proc Natl Acad Sci U S A, 2003
- We have shown that WIP interacts with members of the Wiskott Aldrich syndrome protein  [ **WAS** ]  family and is essential for filopodium formation regulated by  **Cdc42**  GTPase.  Ref: 12724353 J Cell Sci, 2003
- These included small Rho GTPases RhoA, Rac1 and  **Cdc42**  and some of their direct effectors mDia and N WASP  [ **WAS** ] , and also actin binding proteins such as profilin and alpha actinin.  Ref: 12445462 Biochim Biophys Acta, 2002
- A protein designated p145  **was**  identified and shown to be a tyrosine kinase with sequence similarity to mammalian Cdc 42  [ **CDC42** ]  associated tyrosine kinases.  Ref: 11773052 J Biol Chem, 2002
- We show that the endocytic protein, dynamin, competes for binding to the SH3 domains with the neural Wiskott Aldrich Syndrome protein  [ **WAS** ] , an actin filament nucleation protein that is a substrate for activated  **Cdc42** .  Ref: 12686614 Mol Biol Cell, 2003
- These discoveries have fuelled interest in understanding both the role of the actin cytoskeleton as an integral component of lymphocyte activation and the interplay between lymphoid cell cell contact sites immunological synapse, retractile pole structures uropod, distal pole complex, and Rho family GTPases Rac, Rho,  **Cdc42** , their upstream activators Dbl family guanine nucleotide exchange factors and their downstream effectors WASp  [ **WAS** ] , Arp2 3, ADAP.  Ref: 12787750 Curr Opin Immunol, 2003
- These results are consistent with  **Cdc42**  being critically involved in initiating the early events in phagocytosis by its ability to activate Pak and the Wiskott Aldrich Syndrome protein  [ **WAS** ]  that triggers the localized polymerization of actin.  Ref: 12189515 Histochem Cell Biol, 2002

**Non-interactive relationship** (e.g. studied together, co-existance, homology, etc.)

- Coexpression of wild type or activated  **Cdc42**  and N WASP  [ **WAS** ]  also inhibited Golgi to ER transport, but this  **was**  not the case in cells expressing Cdc42V12 and N WASP  [ **WAS** ]  Delta WA, a mutant form of N WASP  [ **WAS** ]  that lacks Arp2 3 binding.  Ref: 11907268 Mol Biol Cell, 2002
- We hypothesize that a transient interaction between C5aR and WASP  [ **WAS** ]  occurs following the stimulation of C5aR and  **Cdc42**  activation.  Ref: 12600272 Biochem J< MedlineTA>Biochem J, 2002
- Active  **Cdc42**  and N WASP  [ **WAS** ]  are recruited to exocytosing cortical granule membranes before F actin coat assembly and coats assemble by  **Cdc42**  dependent, de novo actin polymerization.  Ref: 12872130 Nat Cell BiolNat Cell Biol, 2002
- Interestingly, CD47 ligation failed to induce cell death in mononuclear cells isolated from Wiskott Aldrich syndrome  **WAS**  patients, suggesting the involvement of  **Cdc42**   **WAS**  protein WASP  [ **WAS** ]  signaling pathway.  Ref: 12351399 Blood, 2002
- N WASP  [ **WAS** ]  localizes at the cell periphery, where it controls actin polymerization downstream of signal molecules such as adapter proteins,  **Cdc42** , Src family kinases, and phosphoinositides.  Ref: 12871950 J Biol ChemJ Biol Chem, 2002
- uPA and EGF stimulated chemotaxis, cytoskeletal rearrangements and activation of  **Cdc42** , Rac1 and N WASP  [ **WAS** ]  were studied in the highly metastatic human breast cancer cell line MDA MB 231.  Ref: 11865026 J Cell Sci, 2002
- SMC induced matrix contraction  **was**  markedly blocked 80% by inhibiting the Rho p160ROCK pathway and myosin light chain kinase, and  **was**  decreased to a lesser extent 30 40% by a negative mutant of Rac and inhibitors of phosphatidylinositol 3 kinase PI 3 kinase or p38 mitogen activated protein kinase MAPK, but it  **was**  not affected by the inhibition of Ras and  **Cdc42**  Wiskott Aldrich syndrome protein  [ **WAS** ]  WASP  [ **WAS** ]  pathways.  Ref: 12891007 J Vasc ResJ Vasc Res, 2002
- In this report, the role of the Rho family GTP binding protein  **Cdc42** , in the mastoparan stimulus secretion pathway,  **was**  examined.  Ref: 12135388 Biochemistry, 2002
- The Rho protein  **Cdc42**  induces a significant conformational change in its downstream effector, the Wiskott Aldrich syndrome protein  [ **WAS** ]  WASP  [ **WAS** ] .  Ref: 12680752 Biochemistry, 2003
- The acidic regions of WASp  [ **WAS** ]  and N WASP  [ **WAS** ]  can synergize with CDC42Hs  [ **CDC42** ]  and Rac1 to induce filopodia and lamellipodia.  Ref: 11943145 FEBS Lett, 2002
- The recruitment and activation of WASP  [ **WAS** ]  are coordinated by tyrosine phosphorylated Src homology 2 domain containing leukocyte protein of 76 kDa, which functions as a scaffold, bringing Nck and WASP  [ **WAS** ]  into proximity with Vav 1 and  **Cdc42**  GTP.  Ref: 12874226 J ImmunolJ Immunol, 2002
- While a decrease in the  **Cdc42**  and N WASP  [ **WAS** ]  expression  **was**  also observed, the Rac1 expression remained unchanged.  Ref: 12445462 Biochim Biophys Acta, 2002
- We demonstrate that efficient phosphorylation and dephosphorylation of WASP  [ **WAS** ]  at Y291 are both contingent on binding to activated  **Cdc42** .  Ref: 12769846 Mol Cell, 2003
- Here we show that the Src family kinase Hck induces phosphorylation of WASp  [ **WAS** ]  Tyr 291 independently of  **Cdc42** .  Ref: 12235133 J Biol Chem, 2002
- TCR engagement also results in the assembly of a signaling complex that facilitates the activation of Wiskott Aldrich syndrome protein  [ **WAS** ]  WASP  [ **WAS** ]  by colocalization with  **Cdc42**  GTP.  Ref: 12234365 Immunol Rev, 2002
- The nocodazole effect on microtubule depolymerisation  **was**  partially inhibited through overexpression of Rac,  **Cdc42** , RhoG or RhoA and completely prevented by expression of Vav2.  Ref: 12505155 FEBS Lett, 2003
- We therefore conclude that  **Cdc42**  regulates Golgi to ER protein transport in an N WASP  [ **WAS** ]  dependent manner.  Ref: 11907268 Mol Biol Cell, 2002
- First, we determined that RhoG  **was**  regulated by guanine nucleotide exchange factors that also activate Rac and or  **Cdc42** .  Ref: 12376551 J Biol Chem, 2002
- Also, expression of the verprolin cofilin acidic domain of N WASP  [ **WAS** ] , a downstream  **Cdc42**  effector, in ECs completely interfered with vacuole and lumen formation.  Ref: 11884513 J Cell Sci, 2002
- Phosphoinositide 3 kinase PI3K, small Rho GTPases, such as  **Cdc42**  and Rac1, and neuronal Wiskott Aldrich syndrome protein  [ **WAS** ]  N WASP  [ **WAS** ]  are key effectors that regulate dynamic changes in the actin cytoskeleton and cell migration.  Ref: 11865026 J Cell Sci, 2002
- Tc10 and RhoT as well as  **Cdc42**  bound to the N terminal CRIB motif containing portion of N WASP  [ **WAS** ]  and activated N WASP  [ **WAS** ]  to induce Arp2 3 complex mediated actin polymerization.  Ref: 12456725 J Cell Sci, 2003
- Neuronal differentiation of PC12 and N1E 115 cells induced by dibutyryl cyclic AMP and by serum starvation, respectively,  **was**  prevented by dominant negative  **Cdc42** , Tc10 and RhoT.  Ref: 12456725 J Cell Sci, 2003
- We have shown previously that Wiskott Aldrich syndrome protein  [ **WAS** ]  WASP  [ **WAS** ]  activation at the site of T cell APC interaction is a two step process, with recruitment dependent on the proline rich domain and activation dependent on binding of  **Cdc42**  GTP to the GTPase binding domain.  Ref: 12874226 J ImmunolJ Immunol, 2003
- Second, some effectors of Rac  **Cdc42**  IQGAP2, MLK 3, PLD1, but not others e.g. PAKs, POSH, WASP  [ **WAS** ] , Par 6, IRSp53, interacted with RhoG in a GTP dependent manner.  Ref: 12376551 J Biol Chem, 2002
- Although Rho family GTPases have been implicated in both the invasion and motility of Shigella, the role of  **Cdc42** , an N WASP  [ **WAS** ]  activator, in this process has been controversial.  Ref: 11864577 Curr Biol, 2002
